# Chemical imaging of Fischer-Tropsch catalysts under operating conditions

**DOI:** 10.1126/sciadv.1602838

**Published:** 2017-03-17

**Authors:** Stephen W. T. Price, David J. Martin, Aaron D. Parsons, Wojciech A. Sławiński, Antonios Vamvakeros, Stephen J. Keylock, Andrew M. Beale, J. Frederick W. Mosselmans

**Affiliations:** 1 Diamond Light Source, Harwell Science and Innovation Campus, Didcot, Oxfordshire OX11 0DE, U.K.; 2 Research Complex at Harwell, Harwell Science and Innovation Campus, Didcot, Oxfordshire OX11 0FA, U.K.; 3 Department of Chemistry, University College London, 20 Gordon Street, London WC1H 0AJ, U.K.; 4 ISIS Facility, Rutherford Appleton Laboratory, Harwell Oxford, Didcot, Oxfordshire OX11 0QX, U.K.; 5 Finden Limited, The Workstation Merchant House, 5 East St. Helen Street, Abingdon, Oxfordshire OX14 5EG, U.K.; 6 School of Earth and Environmental Sciences, University of Manchester, Manchester M13 9PL, U.K.

**Keywords:** Operando, multimodal tomography, XRD-CT, XRF-CT, Fischer-Tropsch, Cobalt, intergrowth

## Abstract

Although we often understand empirically what constitutes an active catalyst, there is still much to be understood fundamentally about how catalytic performance is influenced by formulation. Catalysts are often designed to have a microstructure and nanostructure that can influence performance but that is rarely considered when correlating structure with function. Fischer-Tropsch synthesis (FTS) is a well-known and potentially sustainable technology for converting synthetic natural gas (“syngas”: CO + H_2_) into functional hydrocarbons, such as sulfur- and aromatic-free fuel and high-value wax products. FTS catalysts typically contain Co or Fe nanoparticles, which are often optimized in terms of size/composition for a particular catalytic performance. We use a novel, “multimodal” tomographic approach to studying active Co-based catalysts under operando conditions, revealing how a simple parameter, such as the order of addition of metal precursors and promoters, affects the spatial distribution of the elements as well as their physicochemical properties, that is, crystalline phase and crystallite size during catalyst activation and operation. We show in particular how the order of addition affects the crystallinity of the TiO_2_ anatase phase, which in turn leads to the formation of highly intergrown cubic close-packed/hexagonal close-packed Co nanoparticles that are very reactive, exhibiting high CO conversion. This work highlights the importance of operando microtomography to understand the evolution of chemical species and their spatial distribution before any concrete understanding of impact on catalytic performance can be realized.

## Teaser

Multimodal x-ray imaging techniques reveal insight into the structure-function relationships in cobalt Fischer-Tropsch catalysts.

## INTRODUCTION

Unpredictable crude oil prices have stimulated interest in Fischer-Tropsch synthesis (FTS) as an alternative means to catalytically convert synthetic natural gas (“syngas”: CO + H_2_) into functional hydrocarbons to produce sulfur- and aromatic-free fuel. Syngas can be produced by the gasification of coal, methane reforming, or even gasification of biomass, which also renders the FTS process more sustainable. Commercial active catalysts typically comprise iron carbides (high-temperature operation) or metallic cobalt (Co) nanoparticles, where Co tends to produce valuable long-chain hydrocarbons (C_1_ to C_100_) at mild temperatures (200° to 240°C) (*1*–*4*). The Co nanoparticles are supported on high–surface area refractory oxides, such as SiO_2_, Al_2_O_3_, or TiO_2_, and in some cases combinations thereof, to optimize nanoparticle dispersion and stability. The modern-day industrial catalyst has evolved to also contain precious metals [that is, Pt (*5*, *6*), Ru (*7*), and Re (*3*, *8*–*12*)] to “promote” activity, increase lifetime, and particularly control the formation of the most important catalytic component, that is, metal Co nanoparticles. These Co-based multicomponent catalytic systems represent the catalyst of choice for a number of commercial plants throughout the world (Ras Laffan, Qatar; Shell, Bintulu; Velocys, Ohio, etc.).

The successful application of Co FTS technology has been made possible by investment, over many years, in the empirical correlation of catalyst formulation with improved catalytic performance. However, only in the past few years has the understanding of the physicochemical signatures of an active Co FTS catalyst developed (*13*, *14*). This new understanding has evolved mainly as a result of detailed studies of well-defined model catalysts, often investigated using novel x-ray–based catalyst characterization techniques (*15*). Some pertinent observations include the effects of metallic Co particle size on optimal activity and stability (that is, particularly against Co reoxidation). Co nanoparticles below 6 nm, supported on carbon nanofibers, not only exhibit high activity but also display a size dependency for optimal activity (*16*). Above this size, catalytic performance was observed to be size-independent. However, refractory oxides are the support of choice for industrial catalysts, with larger particle sizes (>8 nm) proving to be more stable, selective (to C_5_^+^), and highly active (*17*–*21*). However, although these studies have focused on well-defined catalytic systems, industrial catalysts have a structural complexity beyond the nanoscale, that is, at the microscale and up to the millimeter scale, which needs careful consideration when attempting to translate observations made in the laboratory to industrial operations. For example, when working under mass transfer–limited conditions, a core-shell structure, where Co is preferentially located in the external surface of catalyst grains, favors selectivity to desired long-chain hydrocarbons (*22*). Fine-tuning of process productivity needs to account for significant microstructure features like this. The biggest challenge with commercialization concerns the inability to properly evaluate the evolving chemistry at the microscale and nanoscale with catalytic performance: That knowledge is crucial to improve the accuracy of process models used to optimize the operation of industrial plants.

A key fundamental challenge in the study of heterogeneous catalysts is the location and identification of species responsible for catalytic activity; in particular, it is well known that multicomponent industrial catalysts are heterogeneous in nature and have been recently shown on the nanoscale by Zecevic *et al*. (*23*). The study of catalysts, as they perform the catalytic process in action (that is, under what are termed operando conditions), is essential to understanding their behavior. This is often only possible when using “bright” sources in combination with state-of-the-art detection as available at modern-day synchrotrons. The technical developments at synchrotron sources have seen something of a revolution in recent times with the application of imaging techniques to tackle material problems, with the user nominally in a position to achieve what we recently termed five-dimensional (5D) “color” imaging (that is, where each data set in a tomographic image contains a spectrum or pattern, which provides information on the chemical composition that evolves as a function of time) (*24*). These types of chemical imaging/color imaging techniques have revealed, for example, how metal accumulation on the surface of catalyst particles affects not only the mass transport properties of the material (*25*) but also how it is linked to particle clustering during use and therefore reduced activity (*26*). These techniques have already been used to study Co FTS catalysts under in situ conditions with excellent spatial resolution for x-ray fluorescence computed tomography (XRF-CT) (ca. 30 nm) (*27*). X-ray diffraction computed tomography (XRD-CT) has been used to study catalysts under in situ conditions (*28*, *29*), although with much coarser spatial resolution (>20 μm), revealing the evolution of phases with process conditions, but to date, no studies have collected these multiple modalities simultaneously. Not only does this save time and therefore allow for more data sets to be collected, but it also ensures that all of the information reported is congruent, that is, collected on the same volume of sample under identical conditions, of particular importance when measuring dynamic systems.

By means of advanced x-ray microtomography, we were able to obtain, for the first time, insight into the behavior of the Co active phase, the Ti modified SiO_2_ support, the Re promoter, and the importance of the (co)location of these species. Using standard deposition techniques, we were able to observe how a simple variable such as the sequence of addition of modifiers and metal precursor strongly affects the distribution and structure of the catalyst components and, more importantly, the consequent performance in terms of both activity and selectivity. Here, we used a novel time-resolved “multimodal” x-ray tomography approach (a simultaneous combination of μ-XRD-CT, μ-XRF-CT, and μ-absorption-CT) that enables the effect of chemical distribution and composition of the catalyst, down to 5-μm resolution, to be followed through its lifetime, that is, from the preparation of a preactive state to its evolution into the active state, and because the reaction products were monitored by online mass spectrometry, we were able to put these observations into the context of catalytic performance.

## RESULTS AND DISCUSSION

Two catalyst samples were prepared and loaded into the microreactor (for more details, see figs. S1 and S2 and table S1). One sample follows a conventional impregnation order, in which a silica support was modified with Ti, and in a second step, Co and Re precursors were added. Also, an inverse configuration was prepared, for which the deposition order was swapped.

### Catalyst precursor composition (oxidized sample)

**Fig. 1:**
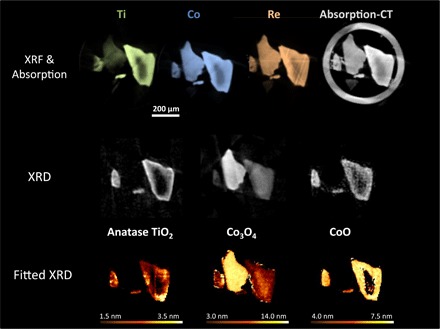
Conventional catalyst precursor structure. (**Top**) XRF-CT reconstructions showing elemental distributions for the conventional catalytic precursor. Green, Ti; blue, Co; orange, Re. Absorption-CT reconstruction (gray) also shows the capillary wall surrounding the particles. (**Middle**) XRD-CT reconstructions of the conventional catalyst revealing the phases present. (**Bottom**) Average crystallite size per pixel for each phase identified. Each pixel is 5 μm × 5 μm. Gas flow was 6 ml min^−1^ He at 25°C.

Before imaging the catalyst under operando conditions, XRF-CT and XRD-CT were conducted on the conventional sample under steady-state conditions; He flow at room temperature. As can be seen in Fig. 1, an uneven distribution of Ti, Co, and Re across the SiO_2_ support is revealed, with a general tendency toward larger concentrations on the outer shell of the catalyst grains for all the elements. The Ti distribution is particularly inhomogeneous and varies significantly both across different catalyst grains and within the same particle. At low Ti loadings, only polymeric Ti–O–Ti species are formed, and no crystalline phase is formed; however, at higher concentrations, XRD-CT data shown in Fig. 1 reveal that the anatase polymorph of TiO_2_ forms with a crystallite size distribution between 1.5 and 3.5 nm. This observation of the very small nano-TiO_2_ can be seen to have two effects on the catalyst deposited on it. First, the Co_3_O_4_ lattice is expanded relative to that on unmodified areas of the support (fig. S3); second, CoO (average crystallite size, ca. 6.0 nm; Table 1) is formed when in close contact with TiO_2_ species. The presence of the CoO phase can be determined by the summed XRD-CT cross section (fig. S4), but only with the combination of XRF/XRD-CT reconstruction can it be correlated to Ti-rich areas. The Co_3_O_4_ has a large crystallite size distribution of 3.0 to 14.0 nm, with the biggest crystallites occurring on the least-modified areas of the support, that is, the lowest Ti concentration, which proves that TiO_2_ aids Co phase dispersion during catalyst preparation. It is likely that the CoO has been formed by the reduction of the outermost layers of the Co_3_O_4_ nanoparticles, in the support interface regions, because the crystallite size in a CoO-free region is much larger than that in areas with CoO (Fig. 1 and figs. S3 and S5A). This poorly crystalline shell also likely accounts for the expansion observed in the lattice spacings of CoO.

**Table 1:** Fitted XRD crystallite sizes and dispersion for Ti and Co phases. The error in the average sizes is ±0.5 nm.

**Sample and condition**		**Phases and average crystallite sizes (nm)**					**Dispersion**	
		**TiO_2_**	**Co_3_O_4_**	**CoO**	**Cubic Co**	**Intergrown Co**	**Cubic Co dispersion**	Intergrown Co dispersion*
Conventional	Calcined	3.0	10.0	6.0	—	—	—	—
	Reduced	3.0	—	—	6.9	3.3	13.9	29.1
	FTS (2 bar)	3.0	—	6.0	9.5	6.5	10.1	14.8
Inverse	Calcined	—	12.5	7.0	—	—	—	—
	Reduced	—	—	—	9.0	3.0	10.7	32
	FTS (2 bar)	—	—	—	8.5	3.0	11.3	32

*Co dispersion was estimated using the formula *D* = 96/*d*, where *D* (%) is Co dispersion and *d* (nm) is Co particle size (*62*), assuming uniform spheres of Co and a site density of 15.2 atoms/nm^2^. If the intergrown and cubic domains are part of a larger particle, then the average particle size will be somewhat larger than that reported, and consequently, the dispersion will be lower.

The Co and Re distribution in the inverse catalyst is quite homogeneous throughout the sample analyzed. However, Ti distribution again shows sharp inhomogeneities (fig. S5A), with the bottom particle in the image exhibiting a severe core-shell effect. These differences in distribution are a direct result of the order of Ti deposition during preparation. When Ti is deposited first, it may hinder subsequent diffusion of Re and Co to the core of the grains. When Ti is deposited second, the Co and Re can freely diffuse throughout the support. As with the conventional catalyst, this high concentration of Ti results in a small relative increase (fig. S3) in the Co_3_O_4_ lattice (average crystallite size, ca. 12.5 nm; Table 1) as well as in the appearance of CoO (average crystallite size, ca. 7.0 nm; Table 1). In this case, the Ti does not form XRD-detectable TiO_2_ crystals but remains in a poorly crystalline state. In addition to this, the reduction to CoO only occurs in the region of the highest Ti concentration, but not in Ti-absent areas, as was the case for the conventional catalyst. It is worth noting that because of the small region of the sample containing CoO, the peaks are not readily identifiable from the summed (bulk) pattern (fig. S4), and it is only through these spatially resolved measurements that it can be identified.

There is no evidence of crystalline Re phases for either catalyst; it has been reported that the Re atoms are dispersed with the Co nanoparticles during the deposition and calcination stages, adopting the cubic Co packing following reduction (*1*, *9*, *30*). The distributions of Co and Re for both samples are highly correlated during all measurement conditions, unlike the more variable Ti distribution. This is expected because Co and Re are incorporated in the same step during preparation. The addition of Re is for the purpose of promoting H_2_ spillover in the initial reduction step to lower the activation temperature.

### Active catalyst

Each reconstructed XRD-CT slice contains 14,641 pixels and therefore a congruent number of diffraction patterns. This number of patterns, along with the challenges posed by the data range and signal-to-noise ratio, prohibits full profile analysis on a per-pixel basis. By performing a cluster analysis, the reconstruction is effectively separated into regions of similar pattern intensity and shape. This massive reduction in data volume and complexity maintains the spatially resolved nature of the measurement, enabling a faster qualitative interpretation of the distribution of phases. Also, a smaller number of patterns facilitate a more detailed analysis of each individual pattern [in this instance, simulation of the diffraction patterns using the DISCUS package (*31*) to aid phase identification and quantification]. These simulations could fit the experimental patterns to a mixture of cubic and intergrown Co (fig. S6 and tables S2 and S3); however, hexagonal Co was not required in the fit—intergrown Co is a mixture of cubic and hexagonal Co, which has undergone stacking faults parallel to the hexagonal 002 plane, reducing the long-range order along the *c* axis of the standard unit cell (*32*). This cluster analysis approach provides a fast, intermediate route to interpreting the XRD-CT data as they are collected, albeit with a lower spatial resolution. To maximize the spatial information, fitting must be done on a pixel-by-pixel basis.

**Fig. 2:**
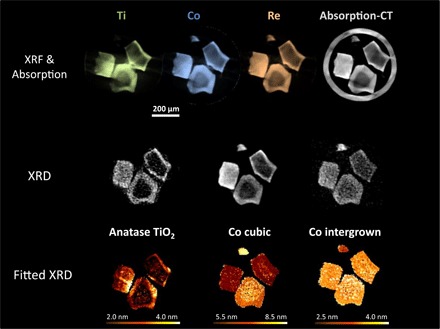
Conventional catalyst structure after reduction. (**Top**) XRF-CT reconstructions showing elemental distributions for the conventional catalyst after the reduction step. Green, Ti; blue, Co; orange, Re. Absorption-CT reconstruction (gray) also shows the capillary wall surrounding the particles. (**Middle**) XRD-CT reconstructions of the conventional catalyst revealing the phases present. (**Bottom**) Average crystallite size per pixel for each phase identified. Each pixel is 5 μm × 5 μm. Gas flow was 6 ml min^−1^ 5% H_2_/He at 400°C.

Following the reduction treatment, both forms of crystalline cobalt oxide are fully reduced, resulting in a mixture of cubic and intergrown metallic Co in both the summed pattern and the XRD-CT slice. There is a decrease in the average Co crystallite size of approximately 35%, consistent with the loss of oxygen from the lattice. The elemental distributions display similar trends to those in the precursor samples (Fig. 2); however, in the regions imaged, the core-shell effect of metal loading for the conventional catalyst is more pronounced in the XRD-CT and XRF-CT reconstructions. Previously, it has been proposed that the smallest CoO crystals cannot be reduced under normal reducing conditions on unmodified SiO_2_ (*33*) when in close contact with the support; however, this is not the case with these promoted catalysts.

The d-spacings for both cubic and intergrown phases indicate that the unit cells undergo less contraction on SiO_2_ supports than on the TiO_2_-modified ones (fig. S7). For the conventional catalyst, the smallest particle has no TiO_2_ present, and both the average crystallite sizes and the d-spacings are larger than those of the particles with anatase content. Similarly, the inverse catalyst (fig. S5B) has no crystalline TiO_2_ on any of the catalyst particles, and the particle sizes and d-spacings are larger than those of the conventional catalyst. Notably, one particle of the inverse catalyst has a very high noncrystalline Ti loading and, in this region, corresponds to slightly smaller crystallite sizes (although with no clear effect on d-spacing of the metallic Co; fig. S8).

The spatial variations in peak width for each cluster (fig. S6 and tables S2 and S3) correspond to the distribution observed by peak fitting on a pixel-by-pixel basis. With both approaches, and for both catalysts, the larger cubic crystallites occur in regions of higher Co loading. Scherrer analysis of the diffraction peaks reveals an average crystallite size of 6.9 nm for cubic Co and 3.3 nm for intergrown Co for the conventional catalyst, and 9.0 nm for cubic Co and 3.0 nm for intergrown Co for the inverse catalyst (Table 1). Given the nature of the stacking faults determined for both phases, and the Co nanoparticle size before reduction, these crystallite sizes are more likely to be sizes of domains within larger particles rather than indicative of the presence of two separate nanoparticles colocated on the support.

### Catalysis: FTS at 2 bar

**Fig. 3:**
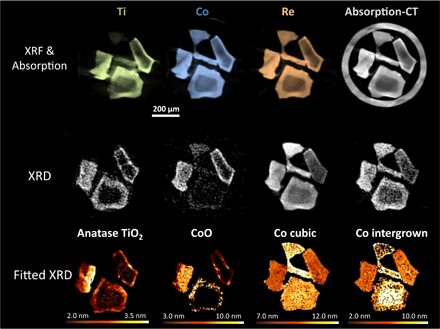
Conventional catalyst structure during FTS. (**Top**) XRF-CT reconstructions showing elemental distributions for the conventional catalyst during FTS at 2-bar pressure. Green, Ti; blue, Co; orange, Re. Absorption-CT reconstruction (gray) also shows the capillary wall surrounding the particles. (**Middle**) XRD-CT reconstructions of the conventional catalyst revealing the phases present. (**Bottom**) Average crystallite size per pixel for each phase identified. Each pixel is 5 μm × 5 μm. Gas flow was 4 ml min^−1^ 5% H_2_/He and 2 ml min^−1^ 5% CO/He at 200°C (H_2_/CO 2:1 and 95% inerts).

Elemental distribution determined by XRF-CT (Fig. 3) is not altered under FTS operation conditions (introduction of CO flow and increase in pressure). However, XRD-CT reveals changes in chemical phase and crystallite size for the conventional catalyst. Local interaction with TiO_2_ has an impact not only on the dispersion of Co phases but also in their structural evolution during FTS operation, because TiO_2_ is known to influence the reducibility profile of Co/SiO_2_ catalysts (*34*). CoO forms in regions of TiO_2_-modified support (and not elsewhere, in any significant quantity) under FTS conditions, with a broad range of crystallite sizes between 4.0 and 8.0 nm. The TiO_2_ size or distribution is not observed to change with the formation of CoO. The largest intergrown crystallites form in Ti-poor areas in the center of the support particles, where the Co concentration is lowest; taking into account the amount of metal present, these larger Co nanoparticles are a minor component of the system and concentrated at the core of the catalyst particles. The XRD simulation results indicate that the increased amount of intergrowth observed is correlated to the reoxidation of cubic Co to CoO and is therefore indicative of an increased proportion of intergrown Co within a particle rather than particle sintering. The smallest Co crystallites are colocated where TiO_2_ and CoO are present, and in regions of significant CoO concentration, the d-spacing is contracted relative to the bulk (fig. S7).

The metallic Co d-spacings exhibit the same trends as for the active sample; however, because the Co intergrown crystallite size has increased, the strain in the 101 reflection is reduced, such that regions closer to the surface of the support (and correlated with TiO_2_/CoO) now have a slightly smaller d-spacing relative to bulk (rather than expanded, as was the case with the Co_3_O_4_ before reduction), indicating a more stable structure. The cubic/intergrown ratio is also decreased, with a larger proportion of intergrown Co than before FTS. This is directly correlated to the formation of CoO, which forms preferentially from cubic Co crystallites, due to the shared unit cell symmetry. This is observed in an asymmetrical change in the peak shape of the cubic 111 reflection (fig. S9); the right-hand side of the peak (high 2θ) decreases in intensity, caused by the thermodynamically favorable oxidation of the smaller cubic Co nanoparticles (*35*, *36*). There is no CoO formed for the inverse catalyst, nor were there any changes in peak symmetry, which is likely a result of having no crystalline TiO_2_ on the support.

The conventional catalyst undergoes an increase in both cubic Co and intergrown average crystallite sizes under FTS conditions (through loss of the smaller crystallites), whereas the inverse catalyst behaves differently, with the Co intergrown phase predominantly still at 3.0 nm and the cubic Co phase at ca. 8.5 nm (Table 1 and fig. S8). Over most of the particles, the Co cubic/intergrown ratio increases; the opposite trend is observed for the conventional catalyst. The one exception to this is in areas of very high Ti loading, where the proportion of cubic Co decreases. This loss of cubic Co may be attributed to some reoxidation to CoO, caused by the high Ti concentration, but not in large enough amounts to crystallize.

Once under FTS conditions, it becomes clear that the degree of stacking faults increases for both catalysts in regions of high Ti concentration. This change is relatively subtle for the inverse catalyst but is very marked for the conventional catalyst. The regions that undergo the greatest increase in stacking faults in the conventional catalyst correspond to regions where CoO has formed following the loss of the smallest cubic Co crystallites. Because these measurements cover 5 μm × 5 μm × 2 μm within the catalyst particles, they consist of an average of all nanoparticles measured within that volume. Therefore, what appears to be a large increase in stacking faults in the high-TiO_2_ region may be due to the loss of the smallest cubic Co nanoparticles (those that reoxidized). If these domains were too small to form significant stacking faults, then their contribution to the voxel would have weighted the average toward a less-disordered structure in the active measurement. However, without nanometric resolution measurements, this cannot be confirmed. What is evident is the correlation between high Ti content, increased stacking faults, and susceptibility toward reoxidation once under FTS conditions. Neither catalyst displayed evidence of carbide, silicate, or titanate phases at any stage during the pretreatment or FTS (*35*, *37*, *38*); however, the time scale of these experiments was shorter than the typical use/lifetime of a catalyst in an industrial plant.

**Fig. 4:**
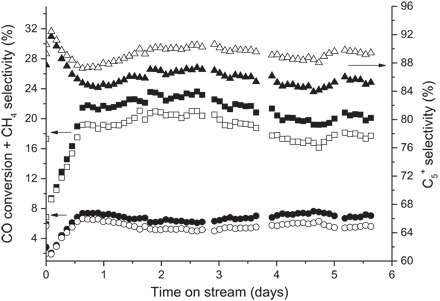
Offline mass spectrometry. ■ □, Offline CO conversion; ● ○, CH_4_ selectivity; C_5_^+^ ▲ △, selectivity. Conventional catalyst (closed points), inverse catalyst (open points). *T* = 205°C, *P* = 20 bar, H_2_/CO = 2;1, 5% N_2_, gas hourly space velocity = 3100 hour^−1^. Tabulated values are shown in table S4.

Operando mass spectrometry results are shown in fig. S10. A comparison of the mass signals before and during FTS provides the best illustration of the generation of products. Both the conventional and inverse catalysts form C_1_^+^-C_4_^+^ fractions, with very minor amounts of C_5_^+^ and C_6_^+^ being generated by the conventional catalyst. The generation of C_4_^+^ confirms that even at a gas pressure of 2 bar, the catalyst was under FTS operating conditions and not merely in situ. The results from the operando mass spectrometry are consistent with those from the offline testing (Fig. 4). Offline catalytic testing highlights the differences in both activity and selectivity between the two catalysts. The conventional configuration yields a 20% CO conversion with 85% selectivity toward C_5_^+^, whereas the inverse configuration is slightly less active (17% CO conversion) and more selective toward the desired products (89%). Those trends are maintained throughout operation. Both catalysts were stable during the 140 hours of offline testing.

### Determining the structure-property relationships

Particle size (*16*–*21*) is an important parameter in determining Fischer-Tropsch activity and selectivity. Co particles that are <6 nm are highly active on carbon supports (*16*), whereas those that are >6 nm are required on refractory oxides to generate the desired longer-chain hydrocarbon products and for stability [for example, the work by Yang *et al*. (*17*) and Fischer *et al*. (*18*)] and have recently been patented (*20*). Support choice has also been demonstrated to play an important role in determining activity (*17*, *18*). Modification with TiO_2_ not only aids the initial reducibility of the catalyst but also causes it to be more susceptible to reoxidation under FTS conditions. The stronger SMSI (strong metal–support interaction) between Co and Ti causes smaller crystallites to form after the calcination and reduction steps, and results in a small expansion in the lattice parameter of the Co_3_O_4_ precursor. Although a degree of sintering appears to occur under FTS conditions for the conventional catalyst, it initially consisted of many smaller cubic Co nanoparticles (less than 6 nm), the loss of which [through reoxidation (*39*)] results in the observed increase in average crystallite size. The extent of reoxidation is directly linked to the TiO_2_ crystallite size on the support. The formation of CoO has been proposed as a deactivation mechanism (*40*)—partly through direct loss of metallic Co and partly due to an increase in water-gas shift activity; however, there is no obvious evidence of water-gas shift from mass spectrometry in this instance.

The superior selectivity to C_5_^+^ of the inverse catalyst is a result of the larger Co crystallites present when compared with those of the conventional catalyst, in agreement with previously published work by Storsæter *et al*. (*21*). The smaller size of the conventional catalyst not only increases the susceptibility toward reoxidation when in close contact with anatase TiO_2_ but also contributes toward its increased selectivity for methane. Here, we directly show that large crystallites are formed as a result of the weaker SMSI in the inverse catalyst. Determination of the “true” active catalyst particle size is complicated by the intergrowth structure, and although electron microscopy would give a direct particle size, doing so under operando conditions presents a very large challenge. If there is also a more local segregation of Re and Co in the inverse catalyst, then the CoO_x_ intermediates during calcination and reduction would be more likely to aggregate, forming larger crystallites. The stronger SMSI in the conventional catalyst prevents this from happening, resulting in smaller crystallites and a more active but less selective (to C_5_^+^) catalyst; with regard to the online mass spectrometry data, the high proportion of inert gas and the large space velocity result in minimal water concentration; therefore, any water-gas or reverse water-gas shift activity would be highly unlikely. Coupled with the offline mass spectrometry data, this suggests that the increased CO conversion observed for the smaller conventional catalyst is not due to water-gas shift effects but to a higher selectivity (*18*, *19*) toward light hydrocarbons (Fig. 4, fig. S10, and table S4).

The similarities in peak positions and intensity between the hexagonal and intergrown phases greatly complicate phase identification by standard methods. In many of the previous studies concerning Fischer-Tropsch catalysts, the characterization was incomplete, and therefore, the significance of the intergrown structure for catalytic activity has never been considered (*41*, *42*). Identification of the presence of intergrown Co, rather than hexagonal Co, aids our interpretation of the nanostructure of the catalyst. It suggests that the active catalyst nanostructure is disordered, with mixtures of cubic and intergrown regions within each Co nanoparticle, rather than being separate cubic/hexagonal close-packed nanoparticles colocated on the support. The simulations reveal a greater degree of stacking faults in the cubic Co phase for the conventional catalyst (20 to 30% versus 15%), with the largest disorder associated with high TiO_2_ content. These stacking faults are distributed throughout the cubic phase, with roughly 1 in 10 layers being faulted. However, in the intergrown regions, they occur every other layer, resulting in a much more disordered structure. Given the lack of hexagonal Co identified in both catalysts, it suggests that the intergrown structure, and not the hexagonal phase, plays a significant role in determining the activity of Co as a Fischer-Tropsch catalyst. In particular, because the intergrown domains in the conventional catalyst are small, they are the likely cause for the high activity to the lighter hydrocarbons, which are less desired for fuel production.

## CONCLUSION

Here, we have reported on the structure of industrially relevant Fischer-Tropsch catalysts under FTS conditions. We have shown how small variations in the deposition sequence can result in catalysts with identical chemical composition, yet result in spatial distribution of elements, different phases, and crystallite sizes, and, more importantly, with different levels of activity and selectivity to specific products. The relationships between these properties are only possible because of the multimodal approach used here to image the catalysts operando. These results represent the first true simultaneous chemical tomography experiment. Several previous studies have been multimodal (*43*–*45*); however, each technique was collected in series, whereas they were collected congruently in this study. Each complete data set took ca. 100 min to collect, with approximately one-third of this time being the readout for the XRD camera. Each XRF spectrum could be collected in 50 ms; however, longer collection times were used to allow synchronization with the XRD collection. With a photon-counting x-ray imaging camera, coupled with recent advances in stage controls on the beamline, one would see the time per data set reduced to ca. 15 min. New scanning methodologies, such as the interlaced method (*46*), can improve the temporal resolution further. One major benefit offered by XRD-CT is the ability to identify and spatially resolve minor components that would otherwise be missed by the corresponding bulk technique. For example, the small CoO contributions to the signal for the conventional catalyst during FTS are not apparent in the summed pattern yet can be readily identified from the reconstruction.

Deposition of the Ti support modifier before the Co catalyst results in smaller, strained Co nanoparticles (a result of a higher degree of intergrowth) that are more active, although they are susceptible to reoxidation in regions of high crystalline Ti loading. The deposition of Ti after Co does not lead to crystalline TiO_2_, potentially due to the Ti depositing on the Co nanoparticles (*47*), and generates larger, less intergrown and therefore more stable Co nanoparticles with greater selectivity toward longer-chain hydrocarbon products. Therefore, this type of engineered physicochemical structure in the catalyst can be used to tune the selectivity of Fischer-Tropsch catalysts, as has already been proposed for zeolites (*48*); this is a previously unconsidered additional dimension to nanoparticle size for optimal catalytic activity. Despite the fact that some of the Co in the conventional catalyst is being reoxidized under FTS conditions (and is therefore expected to be inactive), the activity is maintained, suggesting that the increased intergrowth plays an important role in promoting FTS activity per unit area of metallic Co, most likely by increasing the number of step-edge sites (*49*).

Heterogeneous catalysis is an underpinning technology, playing an increasing role in developing new and more sustainable processes. In addition, it is important to develop atomically efficient/optimized catalysts, but to do this, we need to consider the nanoscale and microscale structure/properties and how these influence catalyst performance. Observing these relationships under process conditions is becoming ever more tractable with developments in state-of-the-art instrumentation and analytical techniques. This approach to analyzing catalysts can pave the way for other catalytic reactions—both gas and liquid phase—to be studied, and, as this study has shown, we are beginning to understand these parameters, which provides us with important new insights that allow us to properly optimize a catalyst for its function.

## MATERIALS AND METHODS

### Catalyst synthesis

Two Co-based model catalysts were supplied by Velocys, with structure and composition chosen to highlight active phase distribution changes in performance. Typically, 15 g of silica (Grace Davison, SG 432; particle size, 180 to 300 μm, pore volume, 1.2 ml g^−1^) was dried in a fan oven at 100°C for 2 hours. The dried support was impregnated at an incipient wetness point with a solution of titanium isopropoxide (Alfa Aesar, 97%) in isopropanol (Fisher, 99.5%). The impregnated support was calcined in a muffle furnace using the following program: ramp at 2°C min^−1^ to 100°C and hold for 3 hours, and then ramp at 2°C min^−1^ to 300°C and hold for 5 hours. Co and Re were then deposited by incipient wetness of an aqueous solution of their precursors, cobalt nitrate hexahydrate (Alfa Aesar, 98%) and perrhenic acid (Sigma-Aldrich, 75 to 85 weight % in H_2_O), respectively. Codeposition of Co and Re (before calcining) ensured thorough mixing and therefore uniform promotion. The sample was calcined following the same program as in the previous step. The resulting “conventional” catalyst had the composition 10% Co/1% Re on 5% TiO_2_/SiO_2_. A second sample was prepared with an “inverse configuration,” where the Ti modification was performed after the incorporation of the metals to the silica. The resulting “inverse” catalyst had the composition 5% Ti/10% Co/1% Re on SiO_2_.

### Offline catalyst characterization

Temperature-programmed reduction (TPR) experiments were carried out using a Micrometrics 2920C instrument. Approximately 250 mg of the sample in a U-shaped quartz tube, with a small wad of quartz wool above and below the sample, was purged with Ar at a flow rate of 50 ml min^−1^ at 150°C for 30 min to remove moisture. The sample was then cooled to 50°C and reduced with 5% H_2_/Ar at a ramp rate of 5°C min^−1^ up to 800°C, under a flow rate of 50 ml min^−1^. H_2_ consumption was monitored by analyzing the outlet gas using an online thermal conductivity detector.

Textural properties were determined from the adsorption-desorption isotherms of nitrogen recorded at 77 K with a Micromeritics TriStar. Specific area was calculated by applying the Brunauer-Emmett-Teller (BET) method to the relative pressure (*P*/*P*_0_) range of the isotherms between 0.03 and 0.3 and taking a value of 0.162 nm^2^ for the cross section of an adsorbed nitrogen molecule at 77 K. Pore size distributions were computed by applying the Barrett-Joyner-Halenda (BJH) model to the desorption branch of the nitrogen isotherms.

For offline testing, the calcined catalysts were loaded into a fixed-bed combinatorial reactor for FTS tests, as described previously (*50*). The catalyst was diluted with SiC and reduced in pure hydrogen at 400°C for 2 hours before testing under industrially relevant FTS conditions (210°C, 20 bar, 2:1 molar ratio H_2_/CO).

### X-ray data collection

A schematic of the experimental setup is shown in fig. S1. The sample (ca. 3 mg) was loaded into a 400-μm OD quartz capillary (20-μm wall thickness) and held in place using quartz wool forming a small packed bed. The capillary was mounted on top of a motorized XY stage to allow for centering of the particle on the axis of rotation. An inlet valve in the base of the capillary mount allowed for gas to be flowed through the capillary, and a side-mounted bracket allowed for the top of the capillary to be sealed; a downstream pressure controller controlled the gas pressure in the capillary. The exhaust gases then flowed into a Pfeiffer OmniStar mass spectrometer fitted with a heated inlet to prevent condensation of products. Gas flow was 6 ml min^−1^ He for the room temperature measurement on the as-received (calcined) samples. Samples were reduced at 400°C under flowing 5% H_2_/He with a ramp rate of 1°C min^−1^. The temperature was then reduced to 150°C at 10°C min^−1^ before the gas mix changed to 5% (2H_2_/CO)/He with a 6 ml min^−1^ flow rate. Last, the temperature was increased to 220°C at 5°C min^−1^ (FTS conditions), and the pressure was increased to 2 bar. Tomographic data were collected on the samples as received (that is, calcined), as reduced, and finally under FTS conditions after a 12-hour equilibration period. Following the measurement at 2 bar, the pressure was increased to 4 bar, and a final set of tomographic measurements was recorded.

Data were recorded at beamline I18 of Diamond Light Source, operating with a Si(111) double-crystal monochromator, with the x-ray beam focused to a 2-μm × 3-μm [full width at half maximum (FWHM)] spot on the sample using Kirkpatrick-Baez mirrors (*51*). XRF-CT data were collected using an incident x-ray energy of 13 keV with a Vortex ME-4 Silicon drift detector and XSPRESS-3 electronics. The sample was rastered across the beam in a translate-rotate data collection scheme, similar to first-generation CT with a pencil beam, with an XRF spectrum collected at 5-μm intervals with a collection time of 1 s per pixel. Typically, 121 translation steps at each rotation angle and 101 rotations (in 2° steps) were used—the exact number of translations was dependent on sample width. XRD-CT data were collected concurrently with the XRF-CT data but with a collection time of 0.6 s per image with a Photonic Sciences CMOS–based x-ray imaging detector. This detector has a longer readout time. Absorption-CT data were collected simultaneously using ion chambers before and after the sample to record the attenuation of x-rays through the sample. This enabled a map of the relative density of the sample to be reconstructed.

### Tomography reconstruction

The absorption-CT was reconstructed first to determine center of mass and rotation corrections (*44*). Data were initially reconstructed using the Savu tomography reconstruction pipeline (*52*–*54*) and additional Python scripts by the filtered back-projection method (*55*), followed by up to 10 refinements using the simultaneous algebraic reconstruction technique (*55*–*57*). The corrections from the absorption-CT reconstructions were applied to the corresponding XRF and XRD reconstructions. This helped to ensure that the reconstructed images from each technique were directly comparable. The regions of interest in the XRF spectrum were windowed before reconstruction to reduce the amount of processing required. The absorption-CT data were then used to correct the fluorescent intensity for sample absorption, taking into account the sample composition, density, and energies of the incident and fluorescent x-rays (*45*, *58*, *59*).

Each XRD image was calibrated (using a LaB_6_ reference material), and data were azimuthally integrated from 2D images to 1D patterns using Dawn v1.9 (*60*). Each 2θ step in the reduced data set was then reconstructed to create the final 3D datastack, having two spatial axes (*x*, *y*) and with the third axis (*z*) being the reconstructed pattern (2θ). The XRD-CT reconstructions were then processed further by (i) fitting a linear baseline across the diffraction peaks of interest to map the location and intensity of the phases of interest and (ii) fitting Gaussian functions to determine the intensity, area, position, and FWHM of the peaks, thereby facilitating the determination of average crystallite size by Scherrer analysis and also any changes in lattice parameters. Principal components analysis and cluster analysis were performed on the reconstructed XRD-CT data using MANTiS (*61*); the resultant patterns from each cluster were then used as the experimental inputs for simulation of the phases present.

### Stacking fault simulations

**Fig. 5:**
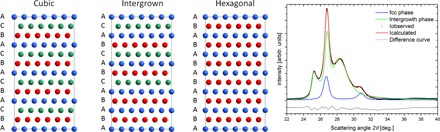
Simulation of diffraction patterns. (**Left**) Co crystal structures used for XRD simulations: Cubic (fcc) with ABC stacking, intergrown with fully random stacking, and hexagonal (hcp) with ABAB layer sequence. (**Right**) Exemplar fit of the XRD pattern generated from cluster analysis of inverse catalyst after reduction using DISCUS. Black points, experimental pattern; red, fitted pattern; blue, fit to cubic phase; green, fit to intergrown phase; gray, difference curve.

Nanoparticles of Co can be obtained in two crystal forms with 3D symmetry: face-centered cubic (fcc), where all layers are stacked in an ABC sequence, and hexagonal close-packed (hcp) with the stacking sequence, ABAB. The stacking direction is [111] for the cubic and [001] for the hexagonal structures. In real structures, nanoparticles of Co may be described as an intergrowth of cubic and hexagonal structures (Fig. 5). The level of mixing of these two phases, commonly referred to as stacking faults, can be quantitatively described as the probability of ABC stacking *p*_STACK_—that is, the probability that the next layer will be translated. A completely cubic structure is denoted by *p*_STACK_ = 0.0, and a completely hexagonal structure is denoted by *p*_STACK_ = 1.0. For any value between 0.0 and 1.0, a variety of intergrowth structures is obtained. For *p*_STACK_ = 0.5, the structure formed consists of randomly stacked layers (the most disordered phase). The cubic and hexagonal structures are three-dimensionally periodic with repetition every three and two layers, respectively. Both structures give sharp Bragg diffraction peaks. However, in the case of a fully random intergrown structure, the translational symmetry is not conserved along the stacking direction, resulting in broader, less intense Bragg diffraction peaks. Simulated x-ray diffraction patterns were compared against those generated by the cluster analysis using the DISCUS package (*31*). The simulated patterns were refined over the range 2.5 Å^−1^ < *Q* < 4.5 Å^−1^, to determine the degree of stacking faults. Two crystal phases with different probabilities of stacking faults were used as inputs: phase 1 with *p*_STACK_ ≈ 0.1, which refers to a cubic structure with 10% probability of hexagonal stacking, and phase 2 with *p*_STACK_ ≈ 0.5, which means a fully intergrowth cubic/hexagonal phase. The XRD patterns were first background subtracted, and later, any additional phases, that is, TiO_2_ (anatase) and CoO, were subtracted where necessary. The final refinement was performed with an evolutionary algorithm implemented in Diffev (*31*). Figure 5 shows an example of a refinement, with contributions from the cubic and intergrown phases shown.

## Supplementary Material

http://advances.sciencemag.org/cgi/content/full/3/3/e1602838/DC1

## SUPPLEMENTARY MATERIALS

Supplementary material for this article is available at http://advances.sciencemag.org/cgi/content/full/3/3/e1602838/DC1 Supplementary Text fig. S1. Schematic of the experimental setup including exemplar XRF spectrum and XRD pattern. fig. S2. TPR results. fig. S3. Deviation in lattice parameter (±1%) from standard value (white) for conventional and inverse catalyst for room temperature measurements of the calcined catalyst. fig. S4. Summed XRD patterns from each XRD-CT measurement. fig. S5A. Inverse catalyst precursor structure. fig. S5B. Inverse catalyst structure after reduction. fig. S5C. Inverse catalyst structure during FTS at 2 bar. fig. S6. Diffraction cluster analysis. fig. S7. Deviation in lattice parameter (±1%) from standard value (white) for conventional catalyst after reduction (top), and during FTS at 2 bar (middle) and 4 bar (bottom). fig. S8. Deviation in lattice parameter (±1%) from standard value (white) for inverse catalyst after reduction (top), and during FTS at 2 bar (middle) and 4 bar (bottom). fig. S9. Change in summed XRD patterns for the conventional catalyst between reduction and FTS (2 bar). fig. S10A. Conventional catalyst mass spectrometry traces for C_1_^+^-C_6_^+^. fig. S10B. Inverse catalyst mass spectrometry traces for C_1_^+^-C_6_^+^. fig. S11. Conventional catalyst structure during FTS at 4 bar. fig. S12. Inverse catalyst structure during FTS at 4 bar. table S1. BET (surface area) and BJH (pore volume and size) results. table S2. Results of phase identification simulations of active reduced catalysts. table S3. Results of phase identification simulations of catalysts under 2-bar FTS conditions. table S4. Activity and selectivity of the catalysts, offline testing corresponding to Fig. 4. References (*63*, *64*)
