# Supplementary material for: Chemical imaging of Fischer-Tropsch catalysts under operating conditions
Source: Sci Adv. 2017 Mar 17;3(3):e1602838. doi: 10.1126/sciadv.1602838 (PMC5357128; doi:10.1126/sciadv.1602838)
Supplement: http://advances.sciencemag.org/cgi/content/full/3/3/e1602838/DC1 [file supp_3_3_e1602838__index.html]

Science Advances | Science Advances

## Supplementary Materials

**This PDF file includes:**

- Supplementary Text
- fig. S1. Schematic of the experimental setup including exemplar XRF spectrum and XRD pattern.
- fig. S2. TPR results.
- fig. S3. Deviation in lattice parameter (±1%) from standard value (white) for conventional and inverse catalyst for room temperature measurements of the calcined catalyst.
- fig. S4. Summed XRD patterns from each XRD-CT measurement.
- fig. S5A. Inverse catalyst precursor structure.
- fig. S5B. Inverse catalyst structure after reduction.
- fig. S5C. Inverse catalyst structure during FTS at 2 bar.
- fig. S6. Diffraction cluster analysis.
- fig. S7. Deviation in lattice parameter (±1%) from standard value (white) for conventional catalyst after reduction (top), and during FTS at 2 bar (middle) and 4 bar (bottom).
- fig. S8. Deviation in lattice parameter (±1%) from standard value (white) for inverse catalyst after reduction (top), and during FTS at 2 bar (middle) and 4 bar (bottom).
- fig. S9. Change in summed XRD patterns for the conventional catalyst between reduction and FTS (2 bar).
- fig. S10A. Conventional catalyst mass spectrometry traces for C1+-C6+.
- fig. S10B. Inverse catalyst mass spectrometry traces for C1+-C6+.
- fig. S11. Conventional catalyst structure during FTS at 4 bar.
- fig. S12. Inverse catalyst structure during FTS at 4 bar.
- table S1. BET (surface area) and BJH (pore volume and size) results.
- table S2. Results of phase identification simulations of active reduced catalysts.
- table S3. Results of phase identification simulations of catalysts under 2-bar FTS conditions.
- table S4. Activity and selectivity of the catalysts, offline testing corresponding to Fig. 4.
- References (*63, 64*)

Download PDF

**Files in this Data Supplement:**

- Adobe PDF - 1602838\_SM.pdf
