# Supplementary material for: Chemical imaging of Fischer-Tropsch catalysts under operating conditions
Source: Sci Adv. 2017 Mar 17;3(3):e1602838. doi: 10.1126/sciadv.1602838 (PMC5357128; doi:10.1126/sciadv.1602838)
Supplement: http://advances.sciencemag.org/cgi/content/full/3/3/e1602838/DC1 [file 1602838_SM.pdf]

Science Advances

AAAS

advances.sciencemag.org/cgi/content/full/3/3/e1602838/DC1

Supplementary Materials for

Chemical imaging of Fischer-Tropsch catalysts under operating conditions

Stephen W. T. Price, David J. Martin, Aaron D. Parsons, Wojciech A. Sławiński, Antonios Vamvakeros, Stephen J. Keylock, Andrew M. Beale, J. Frederick W. Mosselmans

Published 17 March 2017, *Sci. Adv.* **3**, e1602838 (2017)
DOI: 10.1126/sciadv.1602838

This PDF file includes:

- Supplementary Text
- fig. S1. Schematic of the experimental setup including exemplar XRF spectrum and XRD pattern.
- fig. S2. TPR results.
- fig. S3. Deviation in lattice parameter (±1%) from standard value (white) for conventional and inverse catalyst for room temperature measurements of the calcined catalyst.
- fig. S4. Summed XRD patterns from each XRD-CT measurement.
- fig. S5A. Inverse catalyst precursor structure.
- fig. S5B. Inverse catalyst structure after reduction.
- fig. S5C. Inverse catalyst structure during FTS at 2 bar.
- fig. S6. Diffraction cluster analysis.
- fig. S7. Deviation in lattice parameter (±1%) from standard value (white) for conventional catalyst after reduction (top), and during FTS at 2 bar (middle) and 4 bar (bottom).
- fig. S8. Deviation in lattice parameter (±1%) from standard value (white) for inverse catalyst after reduction (top), and during FTS at 2 bar (middle) and 4 bar (bottom).
- fig. S9. Change in summed XRD patterns for the conventional catalyst between reduction and FTS (2 bar).
- fig. S10A. Conventional catalyst mass spectrometry traces for C_1^+-C_6^+.
- fig. S10B. Inverse catalyst mass spectrometry traces for C_1^+-C_6^+.
- fig. S11. Conventional catalyst structure during FTS at 4 bar.
- fig. S12. Inverse catalyst structure during FTS at 4 bar.

- table S1. BET (surface area) and BJH (pore volume and size) results.
- table S2. Results of phase identification simulations of active reduced catalysts.
- table S3. Results of phase identification simulations of catalysts under 2-bar FTS conditions.
- table S4. Activity and selectivity of the catalysts, offline testing corresponding to Fig. 4.
- References (*63*, *64*)

Supplementary Text

Ex situ characterization

Switching the order of deposition of the promoters has no effect on the surface area or the pore size, although the pore volume is less with the TiO_2 modified support (table S1), attributed to the formation of TiO_2 nanocrystals within pores of the SiO_2. Co reduction proceeds via 2 steps; the first is kinetic (Co_3O_4 to CoO), the second thermodynamic (*63*), the addition of the Re promoter facilitates this second step, resulting in the lowered reduction temperature for both catalysts compared with unmodified Co. TPR shows that introducing the Ti modifier in the last step enhances and sharpens the reducibility of Co species (fig. S2), as expected, since the Co interaction is stronger with the TiO_2 modified support, inhibiting the reducibility (*64*).

Operando characterization

In the absorption-CT reconstructions for both catalysts the quartz wool used to hold the particles in place is apparent (as multiple small particles not visible in by XRF or XRD), as well as the capillary wall. The absorption-CT also does not reveal any voids within the support.

Lattice parameters (D-spacings) for both phases have been corrected for thermal expansion at 400 °C for the reduced/active measurement and 200 °C for the FTS measurements (fig. S6 and S7. As is typical for nanoparticles, the cubic 111 d-spacing is contracted relative to the (corrected) bulk value, by ca. 1 %. The intergrown 101 d-spacing on the other hand is ca. 1% larger than the (corrected) bulk value, with the greatest increase directly correlated with the regions with the smallest crystallite sizes. This trend is the opposite of what would ordinarily be expected, i.e. a contraction in lattice parameters with decreasing particle size. An evaluation of the d-spacing change for the cubic 200 and intergrown 100 reflections reveals both are contracted to a similar degree as the cubic 111. However, since the 2nd Co phase is intergrown and not hexagonal the expansion along the 101 d-spacing may be interpreted in terms of the strain induced by the stacking faults as the layers of Co atoms are displaced between cubic and hexagonal packing.

FTS 4 bar

Increasing the pressure to 4 bar causes no observable changes in the elemental or phase distributions during the timescale of the experiment. The crystallite sizes and ratios are nearly identical with those measured at 2 bar. The conventional catalyst crystallite size is ca. 9.5 nm for cubic Co, and a split distribution of 6.5 and 9.5 nm for Co intergrown. The same trends between location, support nature and ratio are also maintained, with an excess of Co intergrown over cubic Co. The inverse catalyst maintains average crystallite sizes of ca. 8.5 nm for cubic Co and 3.0 nm for Co intergrown, along with the higher proportion of Co intergrown.

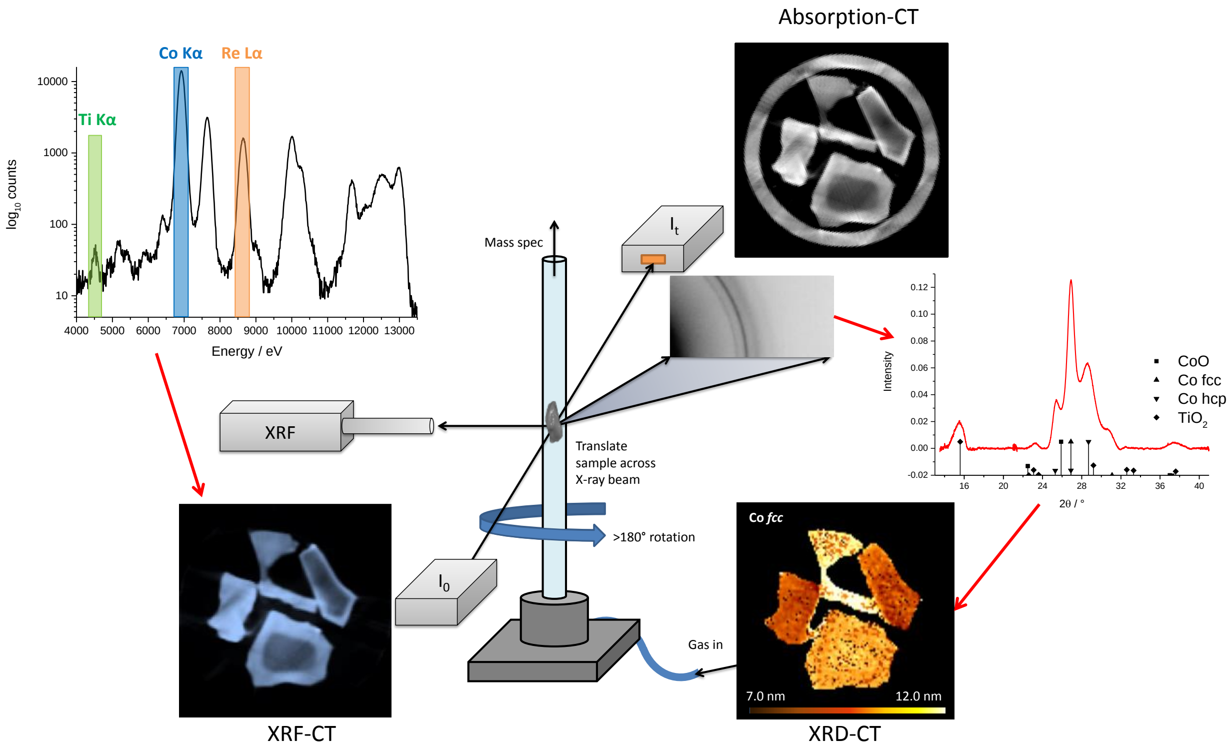

fig. S1. Schematic of the experimental setup including exemplar XRF spectrum and XRD pattern.

table S1. BET (surface area) and BJH (pore volume and size) results. Errors in parentheses.

	Conventional	Inverse
Surface area / $m^2\ g^{-1}$	300 (0.33)	300 (0.33)
Pore volume / $cm^3\ g^{-1}$	0.897 (0.001)	0.916 (0.001)
Pore size / Å	105 (0.16)	104 (0.16)

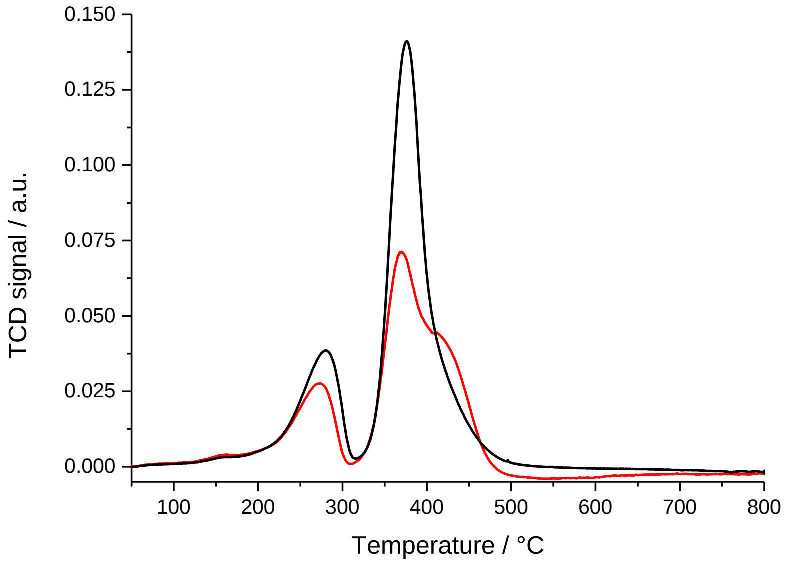

fig. S2. TPR results. Conventional (Ti first, red), Inverse (Ti last, black).

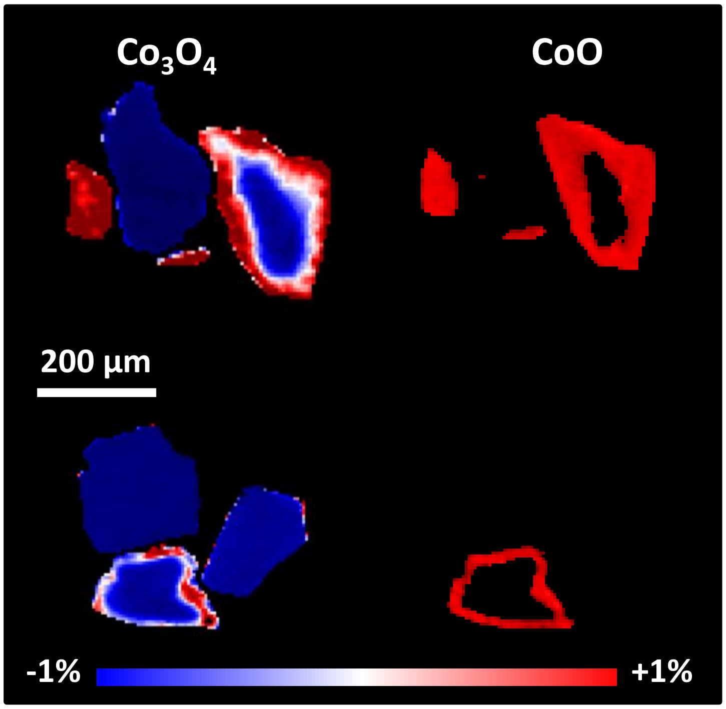

fig. S3. Deviation in lattice parameter (±1%) from standard value (white) for conventional and inverse catalyst for room temperature measurements of the calcined catalyst. Blue corresponds to a 1% contracted lattice, red to a 1% expanded lattice. Co_3O_4 (2.04 Å, 400 reflection) CoO (2.13 Å, 111 reflection). The lattice expansion positively correlates with regions of high anatase TiO_2 concentration.

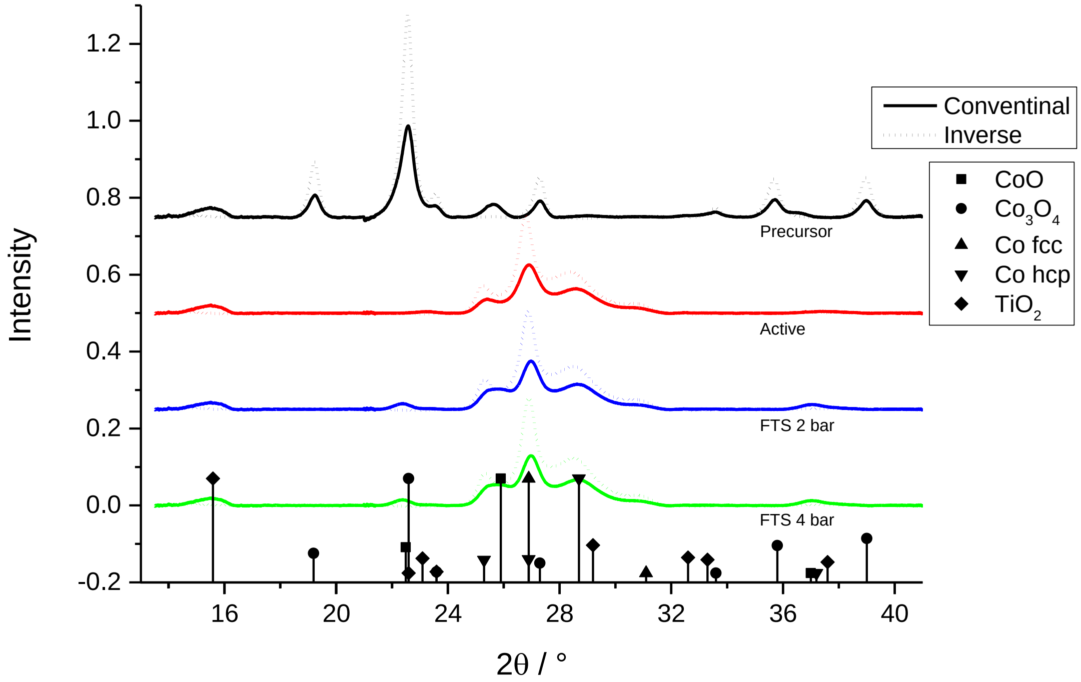

fig. S4. Summed XRD patterns from each XRD-CT measurement.

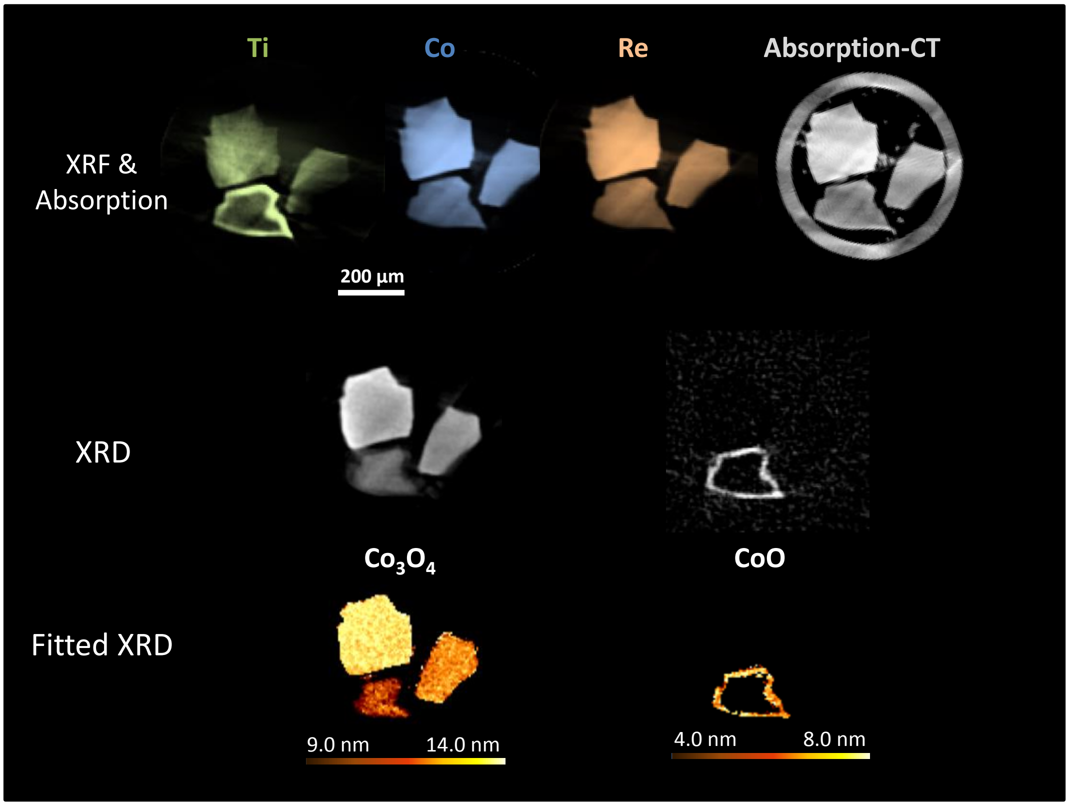

fig. S5A. Top - XRF-CT reconstructions showing elemental distributions for inverse catalyst after calcination step, Ti (green), Co (blue), Re (orange). Absorption-CT reconstruction (grey) also shows the capillary wall surrounding the particles. Middle - XRD-CT reconstructions of the conventional catalyst revealing the phases present. Bottom – Average crystallite size per pixel for each phase identified. Each pixel is 5 x 5 µm. A single crystallite of cubic Co is observed in the reconstruction on one corner of the right hand particle, however this is not apparent from the summed XRD pattern (akin to a bulk measurement).

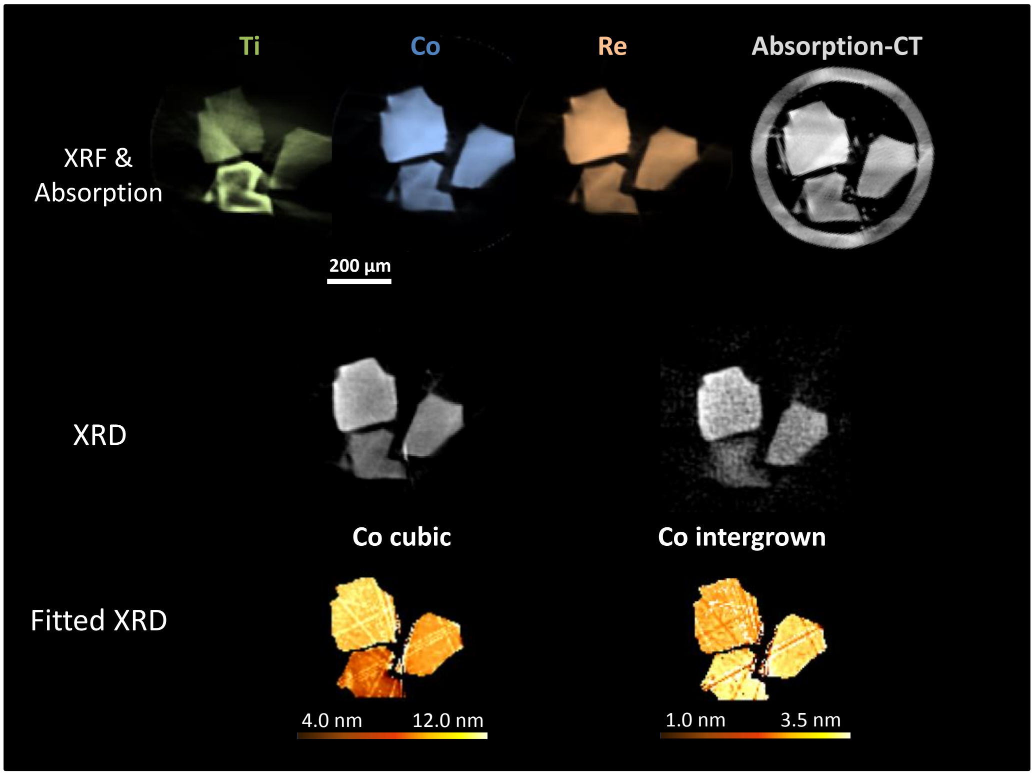

fig. S5B. Top - XRF-CT reconstructions showing elemental distributions for inverse catalyst after reduction step, Ti (green), Co (blue), Re (orange). Absorption-CT reconstruction (grey) also shows the capillary wall surrounding the particles. Middle - XRD-CT reconstructions of the conventional catalyst revealing the phases present. Bottom – Average crystallite size per pixel for each phase identified. Each pixel is 5 x 5 µm. Gas flow was 6 ml min^{-1} 5% H_2/He at 400 °C.

The orthogonal streaks that are present in the fitted XRD of fig. S5b are the result of large crystallites (relative to the beam size). When preferentially oriented with the incident X-ray beam, these large crystallites cause a bright spot in the 2D images (single crystal diffraction vs powder diffraction), which when azimuthally integrated, result in a much sharper diffraction peak than the surrounding area. Since these large crystallites are uncommon on the support (and consequently there are few diffraction spots are observed) there is not enough information to spatially resolve their location during the reconstruction process, causing the appearance of streak artefacts. These artifacts are also observed in figs. S5c, S8 and S12.

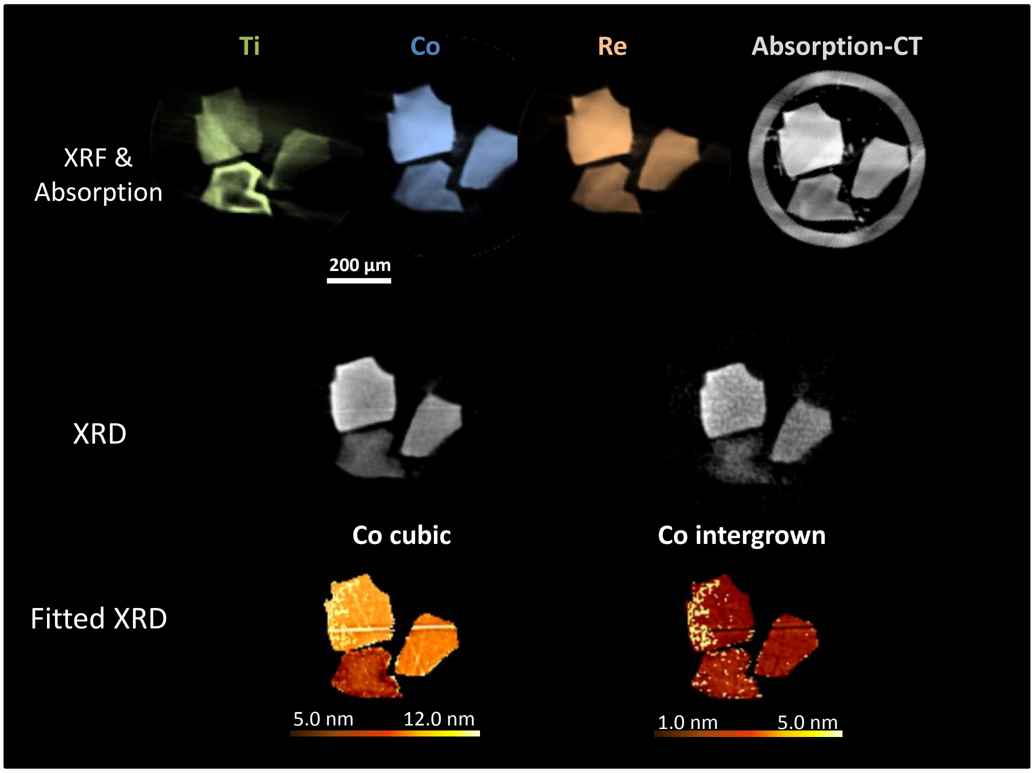

fig. S5C. Top - XRF-CT reconstructions showing elemental distributions for inverse catalyst during FTS at 2 bar pressure, Ti (green), Co (blue), Re (orange). Absorption-CT reconstruction (grey) also shows the capillary wall surrounding the particles. Middle - XRD-CT reconstructions of the conventional catalyst revealing the phases present. Bottom – Average crystallite size per pixel for each phase identified. Each pixel is 5 x 5 µm. Gas flow was 4 ml min^{-1} 5% H_2/He and 2 ml min^{-1} 5% CO/He at 200 °C.

Stacking fault simulation results

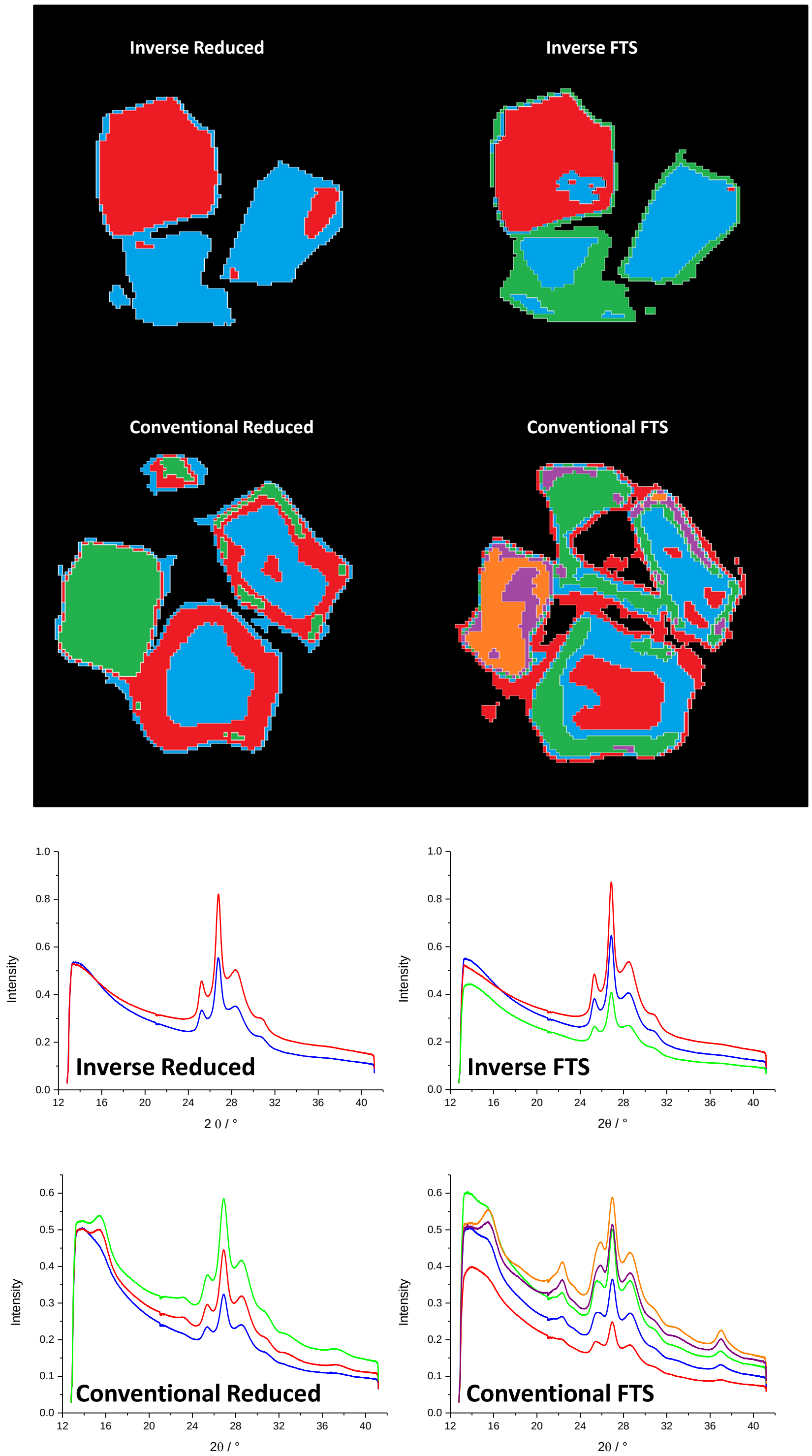

fig. S6. Cluster analysis of XRD-CT reconstruction of conventional and inverse catalysts after reduction and during FTS at 2 bar. The corresponding colored diffraction patterns are the summed patterns from each of the clusters identified. The results of the simulations are in tables S2 and S3.

table S2. Results of phase identification simulations of active reduced catalysts. Font color of cubic Co phase corresponds to region/pattern in fig. S6.

Reduced 400 °C H_2	Sample phase composition [%]		Probability of stacking faults P_{STACK}		FWHM	R value
	Phase 1 Cubic Co	Phase 2 Intergrown Co	Phase 1 Cubic Co	Phase 2 Intergrown Co		
Inverse	15 (2)	85 (2)	0.11	0.56	0.32(1)	0.039
	15 (2)	85 (2)	0.08	0.56	0.25(1)	0.034
Conventional	21 (3)	78 (3)	0.16	0.59	0.54 (1)	0.059
	24 (3)	76 (3)	0.2	0.59	0.53 (1)	0.048
	29 (4)	71 (4)	0.18	0.63	0.59 (2)	0.041

table S3. Results of phase identification simulations of catalysts under 2-bar FTS conditions. Font color of cubic Co phase corresponds to region/pattern in fig. S6.

FTS 200 °C 2:1 H_2:CO 2 bar	Sample phase composition [%]		Probability of stacking faults P_{STACK}		FWHM	R value
	Phase 1 Cubic Co	Phase 2 Intergrown Co	Phase 1 Cubic Co	Phase 2 Intergrown Co		
Inverse	16 (3)	84 (3)	0.11	0.56	0.31 (1)	0.033
	16 (2)	84 (2)	0.10	0.59	0.23 (1)	0.034
	22 (5)	78 (5)	0.20	0.56	0.39 (1)	0.042
Conventional	18 (5)	82 (5)	0.15	0.59	0.49 (1)	0.062
	15 (5)	85 (5)	0.15	0.56	0.41 (1)	0.080
	17 (8)	84 (8)	0.22	0.56	0.40 (1)	0.057
	45 (9)	55 (9)	0.32	0.63	0.47 (2)	0.051
	19 (8)	82 (8)	0.20	0.56	0.46 (2)	0.040

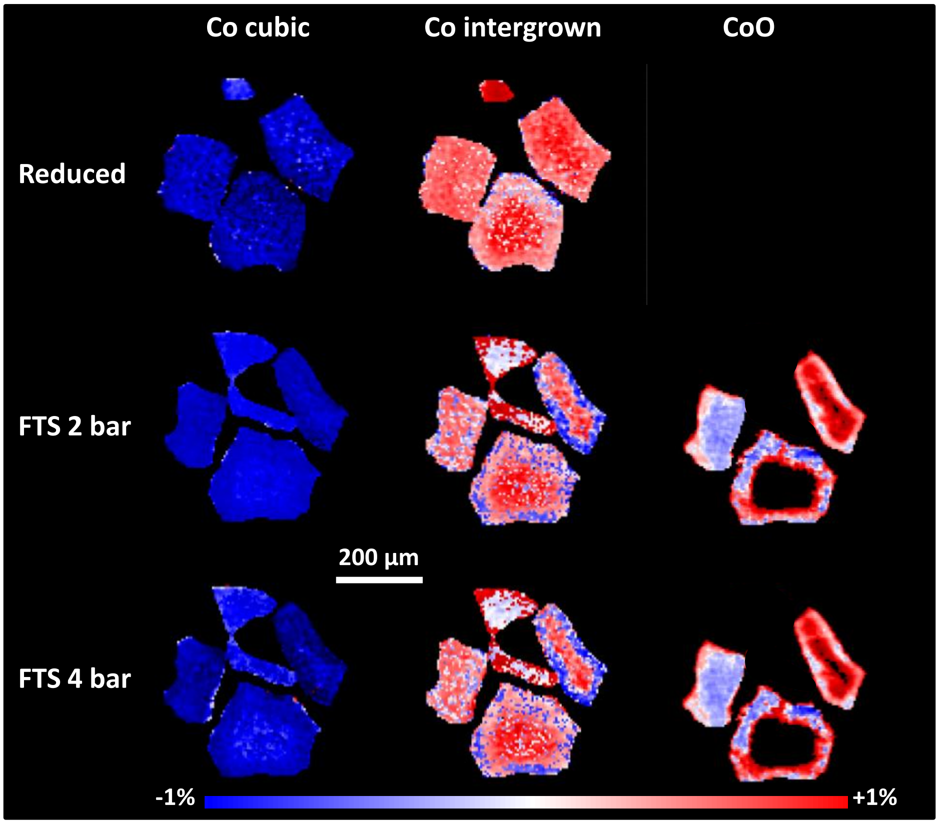

fig. S7. Deviation in lattice parameter (+/- 1%) from standard value (white) for conventional catalyst after reduction (top), and during FTS at 2 bar (middle) and 4 bar (bottom). Blue corresponds to a contracted lattice, red to an expanded lattice. Cubic Co (2.05 Å, 111 reflection) Co intergrown (1.92 Å, 101 reflection), CoO (2.13 Å, 111 reflection). Lattice parameters have been corrected by the thermal expansion coefficient of Co (13x10-6 K^{-1}) so that the values shown are deviations from the lattice parameters at the temperature of measurement (i.e. 400 °C for reduction) and not the standard lattice parameters.

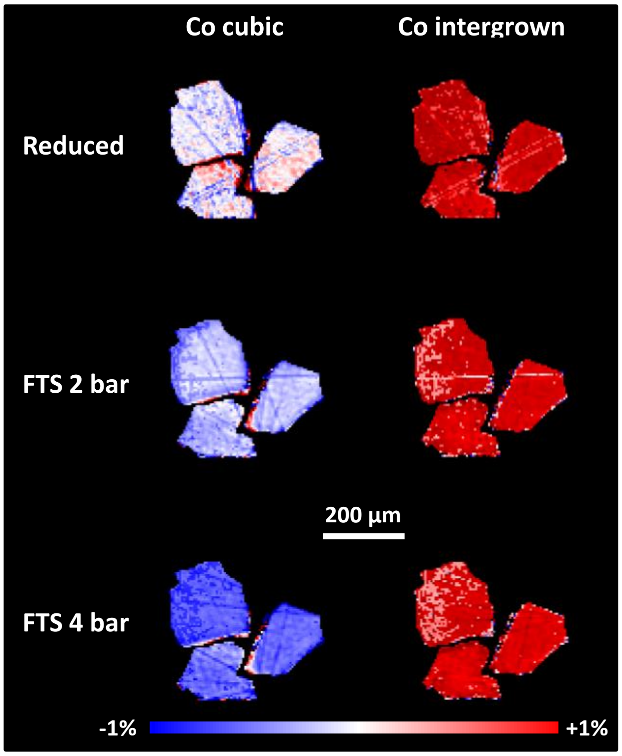

fig. S8. Deviation in lattice parameter (+/- 1%) from standard value (white) for inverse catalyst after reduction (top), and during FTS at 2 bar (middle) and 4 bar (bottom). Blue corresponds to a contracted lattice, red to an expanded lattice. Co cubic (2.05 Å, 111 reflection) Co intergrown (1.92 Å, 101 reflection). Ratio of cubic: intergrown (intensity of Co cubic 111 : Co intergrown 101) is also shown. Lattice parameters have been corrected by the thermal expansion coefficient of Co ($13x10^{-6}$ K^{-1}) so that the values shown are deviations from the lattice parameters at the temperature of measurement (i.e. 400 °C for reduction) and not the standard lattice parameters.

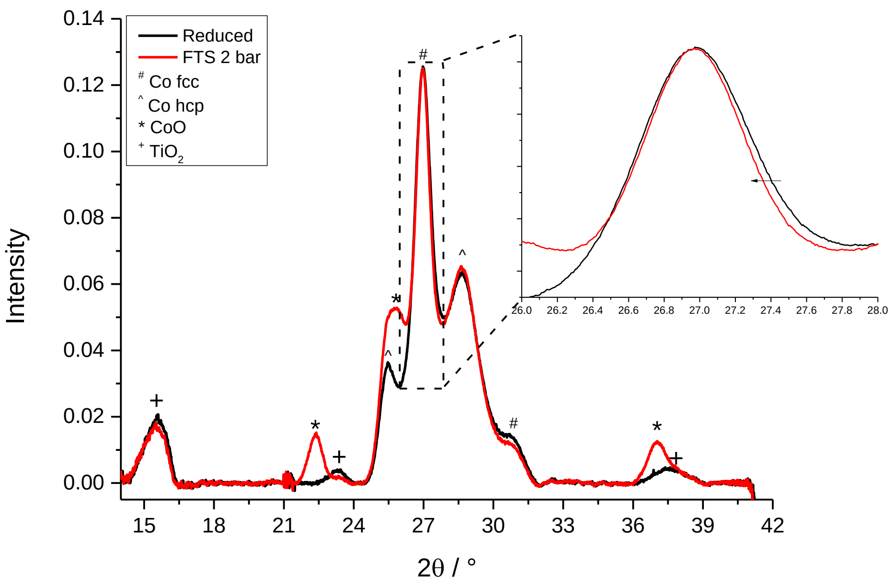

fig. S9. Change in summed XRD patterns for the conventional catalyst between reduction and FTS (2 bar). As well as formation of CoO (*), the right hand slope of the peak at 26.9 ° shifts to a lower 2θ value indicating a loss of the smallest cubic crystallites. NB the 2θ scale for the reduced measurement has been expanded to account for the temperature difference between the 2 measurements to facilitate the visualization of the asymmetric peak shape change.

Operando mass spectrometry

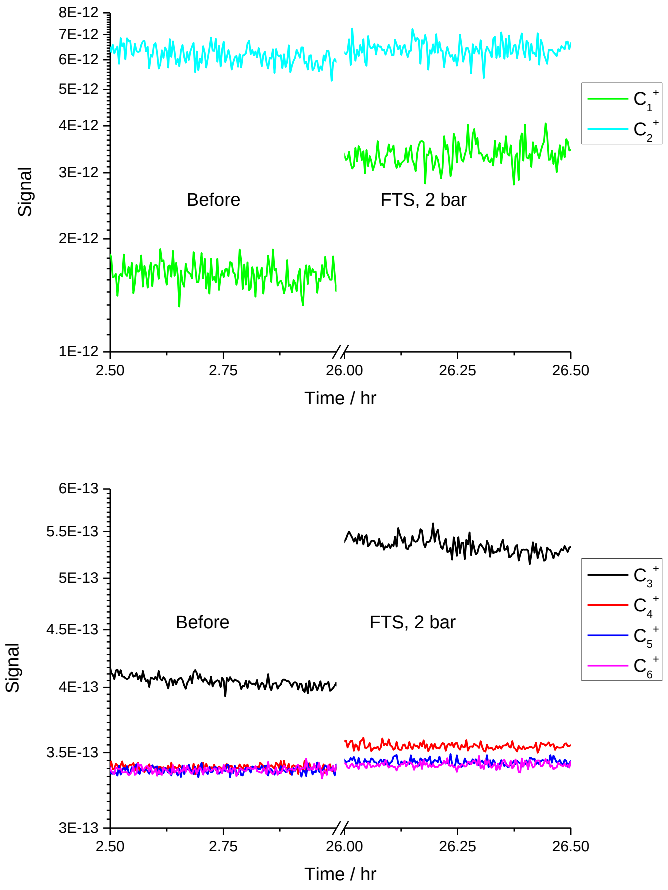

fig. S10A. Conventional catalyst mass spectrometry traces for C_1^+-C_6^+. Comparison of before and during FTS at 2 bar gas pressure (without and with CO flow).

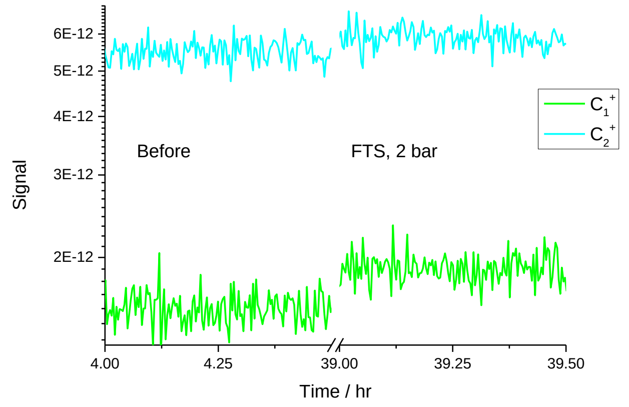

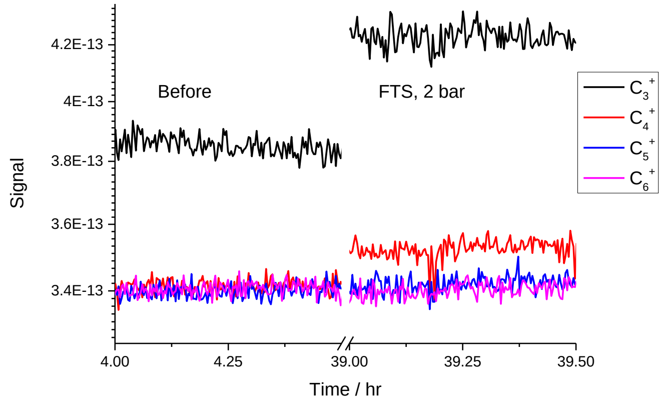

fig. S10B. Inverse catalyst mass spectrometry traces for C_1^+-C_6^+. Comparison of before and during FTS (without and with CO flow).

table S4. Activity and selectivity of the catalysts, offline testing corresponding to Fig. 4.

Catalyst	CO Conversion (%)		Rate of deactivation (% day^{-1})	C_5^+ selectivity (%)		CH_4 selectivity (%)	
	24 h	140 h		24 h	140 h	24 h	140 h
Conventional	21.7	19.8	-0.75	84.9	85.0	7.2	7.2
Inverse	19.2	17.3	-0.81	87.9	89.2	6.3	5.8

It should be emphasized that these materials are model catalysts optimized for x-ray penetration depth with a lower than optimal Co loading. Consequently, the activity for both of catalysts is lower than for those with a more typical Co loading, e.g. 40%, under the stringent reaction conditions.

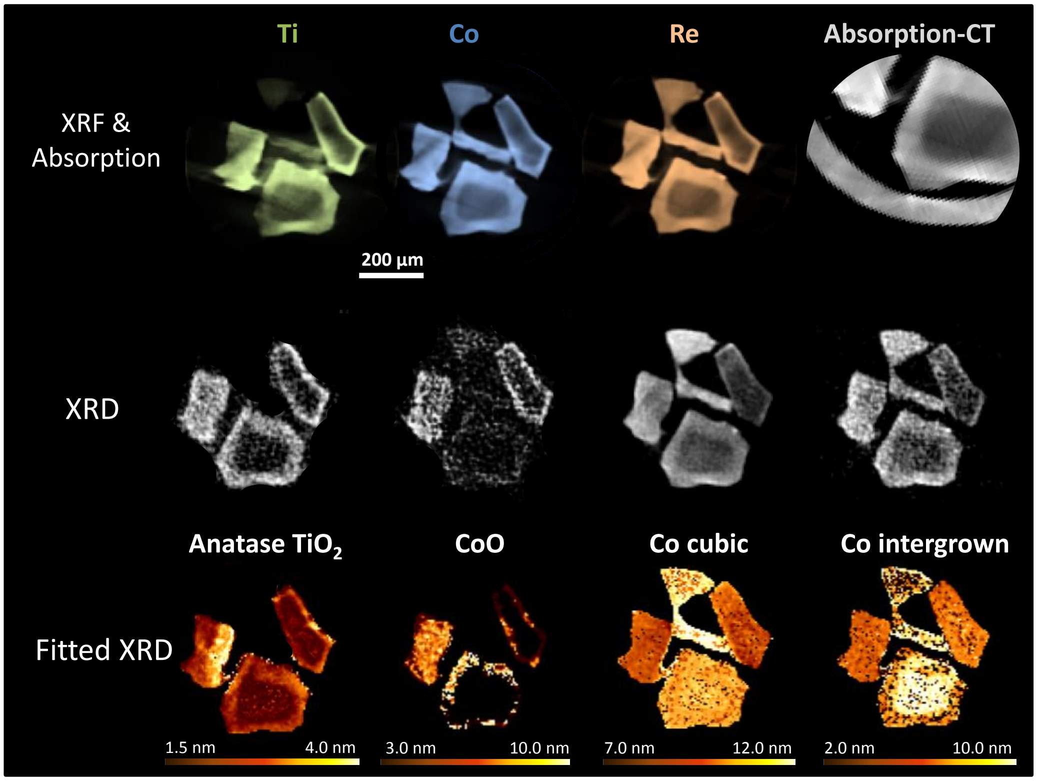

fig. S11. Top - XRF-CT reconstructions showing elemental distributions for conventional catalyst during FTS at 4 bar, Ti (green), Co (blue), Re (orange). Absorption-CT reconstruction (grey) also shows the capillary wall surrounding the particles. Middle - XRD-CT reconstructions of the conventional catalyst revealing the phases present. Bottom – Average crystallite size per pixel for each phase identified. Each pixel is 5 x 5 µm. Gas flow was 4 ml min^{-1} 5% H_2/He and 2 ml min^{-1} 5% CO/He at 200 °C.

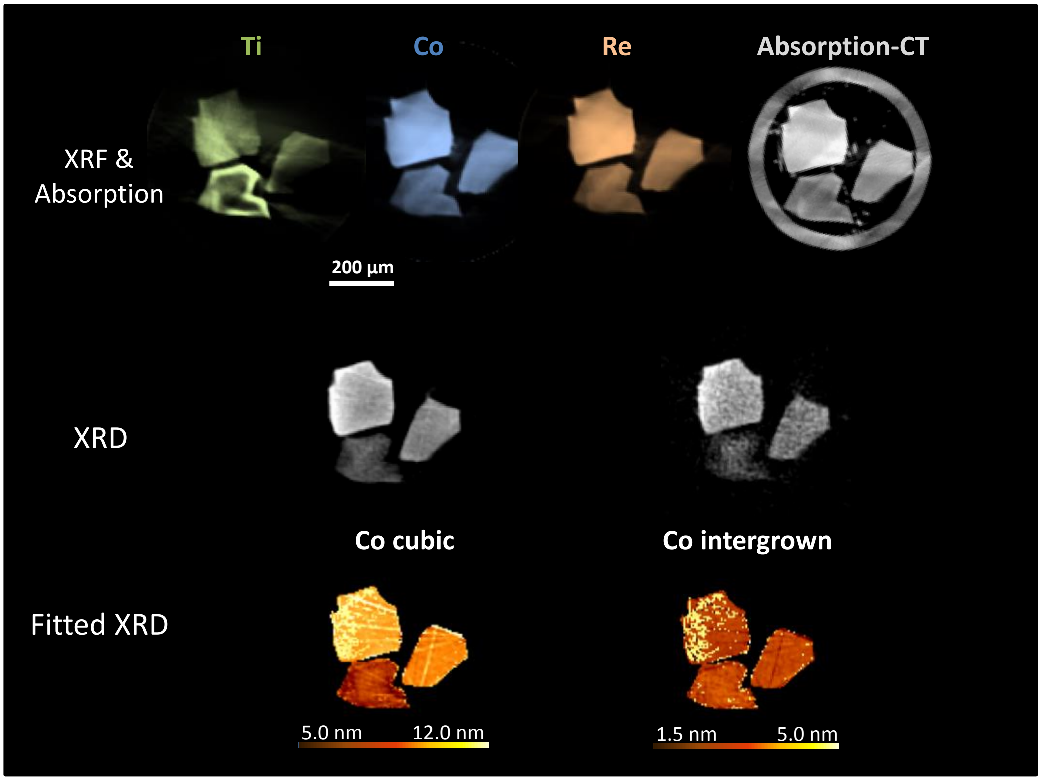

fig. S12. Top - XRF-CT reconstructions showing elemental distributions for inverse catalyst during FTS at 4 bar, Ti (green), Co (blue), Re (orange). Absorption-CT reconstruction (grey) also shows the capillary wall surrounding the particles. Middle - XRD-CT reconstructions of the conventional catalyst revealing the phases present. Bottom – Average crystallite size per pixel for each phase identified. Each pixel is 5 x 5 µm. Gas flow was 4 ml min-1 5% H2/He and 2 ml min-1 5% CO/He at 200 °C.
